# Phosphorus-Doped Carbon Nitride Materials for Enhanced Photocatalytic Degradation of Organic Pollutants under Visible-Light Irradiation

**DOI:** 10.1021/acsomega.5c04783

**Published:** 2025-09-08

**Authors:** Yi-Zhen Huang, Yu-Shen Lin, Yu-Shan Lin, Tai-Chia Chiu, Cho-Chun Hu

**Affiliations:** Department of Applied Science, 63285 National Taitung University, No. 369, Sec. 2, University Road, Taitung 95092, Taitung, Taiwan, R.O.C.

## Abstract

In this study, phosphorus-doped graphitic carbon nitride (P-doped g-C_3_N_4_, denoted PCN) was synthesized via thermal polymerization. Phosphorus doping significantly enhanced the photocatalytic efficiency by improving light absorption capabilities and promoting charge carrier separation. This photocatalyst was used for removing persistent antibiotics, such as trimethoprim (TMP), from aquatic environments. TMP’s high photostability necessitates effective treatment strategies. The optimized catalyst, 0.1 PCN, achieved over 99% degradation of TMP within 90 min under 405 nm LED irradiation. Mechanistic investigations identified singlet oxygen (^1^O_2_) and superoxide radicals (·O_2_^–^). Moreover, 0.1 PCN demonstrated excellent stability and recyclability across multiple operational cycles, maintaining high degradation efficiency even in a complex matrix such as tap water and lake water. This research highlights the significant potential of P-doped g-C_3_N_4_ as an effective, sustainable, and metal-free photocatalyst for the removal of antibiotic contaminants from water.

## Graphical abstract

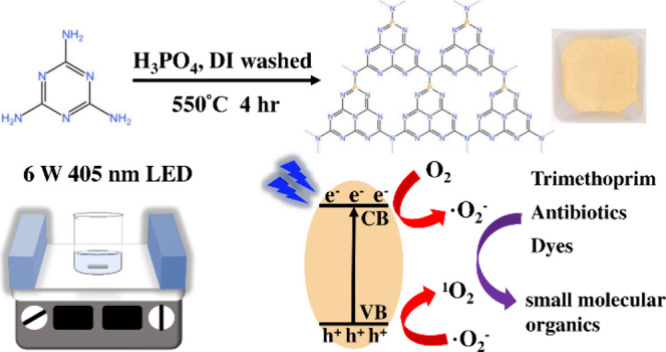

## 1 Introduction

Global population growth and industrialization have led to increased energy demands and environmental pollution, highlighting the need for sustainable energy sources and effective environmental remediation strategies. Among emerging contaminants, antibiotics released into aquatic ecosystems pose significant threats to ecological balance and human health.^1^ Trimethoprim (TMP), a widely used broad-spectrum antibiotic in human and veterinary medicine, is of particular concern. A significant fraction (approximately 80%) of administered TMP is excreted unmetabolized, primarily entering waterways via effluent discharge.^2^ The persistence and widespread detection of TMP and similar residues in wastewater, surface water, and even drinking water sources (at ng/L to μg/L levels) is a global issue.^3^ Such contamination presents risks to aquatic life and may contribute to the spread of antimicrobial resistance. Conventional wastewater treatments, such as activated sludge, are often ineffective in removing these recalcitrant molecules. Furthermore, TMP exhibits high photostability under solar irradiation, hindering its natural degradation in aquatic environments.^4^ Consequently, there is an urgent need for advanced technologies capable of effectively removing TMP from water matrices.

Various methodologies have been explored for the removal of organic pollutants from water, with oxidative degradation and adsorptive processes attracting significant interest. Oxidative strategies, including photocatalysis, Fenton/photo-Fenton reactions, and sonolysis, can convert persistent organic contaminants into less harmful products.^5–8^ Among these advanced oxidation processes (AOPs), photocatalysis has emerged as particularly promising due to its high efficiency, environmental benignity, and low tendency to generate secondary pollutants.^9^ Owing to its high cost-effectiveness, operational simplicity, nontoxicity, and ability to function under mild conditions, photocatalysis has been widely applied for the treatment of various pollutants, such as antibiotics, pesticides, and personal care products.^10–13^ Consequently, correct research focused on developing next-generation photocatalysts that combine high activity, long-term stability, and sustainability to meet the demands of advanced water purification.

Graphitic carbon nitride (g-C_3_N_4_), a metal-free polymeric semiconductor, has attracted considerable attention for energy conversion and environmental applications, including photocatalytic hydrogen evolution and contaminant degradation.^14–17^ Its appeal stems from favorable properties such as facile synthesis, high thermal/chemical stability, a suitable electronic band structure, and low cost using abundant precursors.^18–20^ Despite these merits, bulk g-C_3_N_4_ suffers from limitations that hinder its photocatalytic efficiency, including low specific surface area, limited visible-light absorption, poor conductivity, and rapid recombination of photogenerated charge carriers.^21–23^ Heteroatom doping has emerged as a simple yet highly effective strategy to overcome these limitations and enhance the photocatalytic performance of g-C_3_N_4_. For instance, Kang and co-workers showed that phosphorus-doped g-C_3_N_4_ displayed substantially improved activity for 2,4-dichlorophenoxyacetic acid (2,4-D) degradation compared to g-C_3_N_4_.^24^ Matějka et al. found that phosphorus doping using hexachlorocyclotriphosphazene (HCCP) markedly enhanced rhodamine B (RB) degradation.^25^ These studies demonstrate the effectiveness of heteroatom doping for modulating the physicochemical properties of g-C_3_N_4_ and boosting its performance in environmental applications.

Herein, we report the synthesis of phosphorus-doped g-C_3_N_4_ via a facile thermal polymerization method, designed to selectively oxidize persistent organic pollutants through photosensitized singlet oxygen (^1^O_2_) generation. The photocatalytic performance was evaluated using TMP as a model pharmaceutical contaminant, with degradation efficiency assessed through kinetic studies and reactive oxygen species (ROS) identification. Notably, the degree of structural exfoliation and the resulting photocatalytic activity were affected by the variation of the H_3_PO_4_ concentration used during the synthesis. This tunable approach offers a practical and potentially scalable route to high-performance photocatalysis, contributing to the rational design of sustainable materials for advanced water treatment.

## 2 Experimental Section

### 2.1 Materials

Melamine (99%), trimethoprim (TMP, ≥98%), sulfamethazine (SMZ), sulfamethoxazole (SMX), tetracycline (TC), chlortetracycline (CTC), oxytetracycline (OTC), doxycycline (DC), methylene blue (MB), rhodamine B (RB), crystal violet (CV), methyl orange (MO), sodium nitrate, ammonium oxalate monohydrate (AO), l-histidine (l-His), 1,4-benzoquinone (p-BQ), ammonium ethanoate, and sodium sulfate were purchased from Sigma-Aldrich (St. Louis, MO, USA). Phosphoric acid (85%) and formic acid (≥98%) were purchased from Honeywell (Charlotte, NC, USA). Methanol (HPLC grade) was purchased from Spectrum Chemical (New Brunswick, NJ, USA). Isopropanol (IPA, ACS grade) and acetonitrile (ACN, HPLC grade) were purchased from J. T. Baker (Phillipsburg, NJ, USA). Nafion powder was purchased from TRC (Toronto, ON, Canada). Deionized (DI) water was used throughout the experiments. All chemicals were used as received without further purification.

### 2.2 Synthesis of g-C_3_N_4_ (CN)

Pristine graphitic carbon nitride (g-C_3_N_4_, denoted as CN) was synthesized via thermal polymerization. Typically, 5.0 g of melamine was placed in a crucible with a lid and calcined at 550 °C for 4 h in a static air atmosphere using a muffle furnace (heating rate, typically, 5 °C min^–1^). The resulting pale orange–yellow powder was collected after cooling naturally to room temperature.

### 2.3 Synthesis of Phosphorus-Doped g-C_3_N_4_ (PCN)

Phosphorus-doped g-C_3_N_4_ samples were prepared via a modified thermal polymerization method. In a typical synthesis, 5.0 g of melamine was added to 10 mL of H_3_PO_4_ solution (0.1, 0.2, or 0.5 M) in a 100 mL beaker under vigorous magnetic stirring (200 rpm). The suspension was stirred continuously for 2 h at room temperature. The resulting mixture was washed thoroughly with DI water via centrifugation until the supernatant was neutral and then dried at 60 °C overnight. The dried precursor was subsequently calcined under the same conditions used for CN (550 °C for 2 h in air). The collected pale orange–yellow powders were designated as 0.1, 0.2, and 0.5 PCN, corresponding to the initial H_3_PO_4_ concentrations used.

### 2.4 Characterization

Sample morphologies were examined by using a field emission scanning electron microscope (FESEM; JEOL JSM-7800F). Powder X-ray diffraction (XRD) patterns were recorded by using a low-temperature X-ray diffractometer (Bruker, D8 Discover X-ray Diffraction System). X-ray photoelectron spectroscopy (XPS) was performed on a Thermo Scientific K-Alpha instrument. Binding energies were calibrated using the adventitious C 1s peak at 284.8 eV. UV–vis diffuse reflectance spectra (DRS) were obtained by using a Specord 210 plus UV–vis spectrophotometer (Analytik Jena, Germany). Photoluminescence (PL) spectra were recorded on an RF-6000 fluorescence spectrometer (Shimadzu, Japan) using an excitation wavelength of 380 nm.

### 2.5 Photocatalytic Activity Evaluation

Photocatalytic degradation experiments were performed in a cylindrical photoreactor using TMP as the target pollution. In a typical run, 50 mg of photocatalyst powder was suspended in 50 mL of an aqueous TMP solution (100 μM). The suspension was magnetically stirred (200 rpm) in the dark for 30 min to ensure adsorption–desorption equilibrium before illumination. The light source consisted of two 3 W 405 nm LEDs positioned symmetrically 7.1 cm from the reactor walls. During irradiation, 4 mL aliquots were withdrawn at 15 min intervals. The withdrawn samples were immediately centrifuged (10,000 rpm, 10 min) and filtered through a 0.22 μm syringe filter (PTFE) to remove catalyst particles. The concentration of residual TMP was determined by high-performance liquid chromatography (HPLC; ECOM) coupled to a UV–vis detector (Agilent 1260 Infinity) set at 271 nm. Separation was achieved using an Agilent Poroshell 120 SB-C18 column (2.1 × 100 mm, 2.7 μm) maintained at 40 °C. The mobile phase consisted of acetonitrile and 0.1% formic acid solution (containing 5.06 mM ammonium acetate) at a volume ratio of 8:92 (v/v). The flow rate was 0.3 mL/min. The degradation percentage was calculated using eq 1:

$$\mathrm{Degradation}\;(\%)=[(C_{0}-C_{t})/C_{0}]\times 100$$ 1

where *C*_0_ is the initial TMP concentration after adsorption equilibrium, and *C*_*t*_ is the concentration at time *t*.

Degradation intermediates were analyzed using liquid chromatography–mass spectrometry (LC-MS; Shimadzu, LC-MS-8060) equipped with the same HPLC column and similar mobile-phase conditions. The environmental risks of TMP and its degradation intermediates were predicted by the quantitative structure–activity relationship (QSAR) method utilizing the Toxicity Estimation Software Tool (T.E.S.T.).

### 2.6 Scavenger Tests

To elucidate the primary reactive species involved in the TMP degradation, scavenger experiments were conducted under conditions identical to those for the photocatalytic test, with the addition of specific scavengers prior to illumination. Isopropanol (IPA, 0.1 M), sodium nitrate (0.1 M), ammonium oxalate (AO, 0.1 M), l-histidine (l-His, 0.1 M), and p-benzoquinone (p-BQ, 0.1 M) were used scavengers for hydroxyl radicals (·OH), electrons (e^–^), holes (h^+^), singlet oxygen (^1^O_2_), and superoxide radicals (·O_2_^–^), respectively.

### 2.7 Photoelectrochemical Measurements

Electrochemical impedance spectroscopy (EIS) and other photoelectrochemical measurements were performed using a Zive SP1 electrochemical workstation (ZiveLab) in a standard three-electrode quartz cell. A photocatalyst-coated fluorine-doped tin oxide (FTO) glass substrate served as the working electrode, a platinum wire served as the counter electrode, and a Ag/AgCl electrode (saturated KCl) served as the reference electrode. A 0.5 M Na_2_SO_4_ aqueous solution served as the electrolyte. To prepare the working electrode, 20 mg of the photocatalyst was dispersed in 2 mL of ethanol containing 10 μL of 5 wt % Nafion solution. The mixture was sonicated for 30 min to form a uniform slurry, and 400 μL was drop-cast onto a cleaned FTO substrate (2 × 2 cm^2^) and dried at 60 °C overnight.

## 3 Results and Discussion

### 3.1 Structural and Chemical Characterization

The morphological characteristics of the CN and PCN samples were investigated by using scanning electron microscopy (SEM). As shown in Figure 1a, the pristine CN sample exhibited a typical stacked, layered structure characteristic of bulk g-C_3_N_4_. In comparison, treatment with H_3_PO_4_ induced a significant morphological transformation (Figure 1b–d). The compact structure of CN collapsed, yielding irregular, loosely aggregated layers in the PCN samples, suggesting that the treatment promotes the exfoliation of the bulk precursor. Among the treated samples, 0.1 PCN (prepared using 0.1 M H_3_PO_4_) displayed the most pronounced fragmentation and flake-like morphology, indicating the most efficient exfoliation under these conditions. Furthermore, the surfaces of the PCN materials appeared to be porous. This morphological change, as widely supported by the literature, would significantly increase the specific surface area and enhance porosity, thereby providing more active sites and improving mass transfer for pollutants, which contribute to their photocatalytic performance.^26^

**Figure 1:**
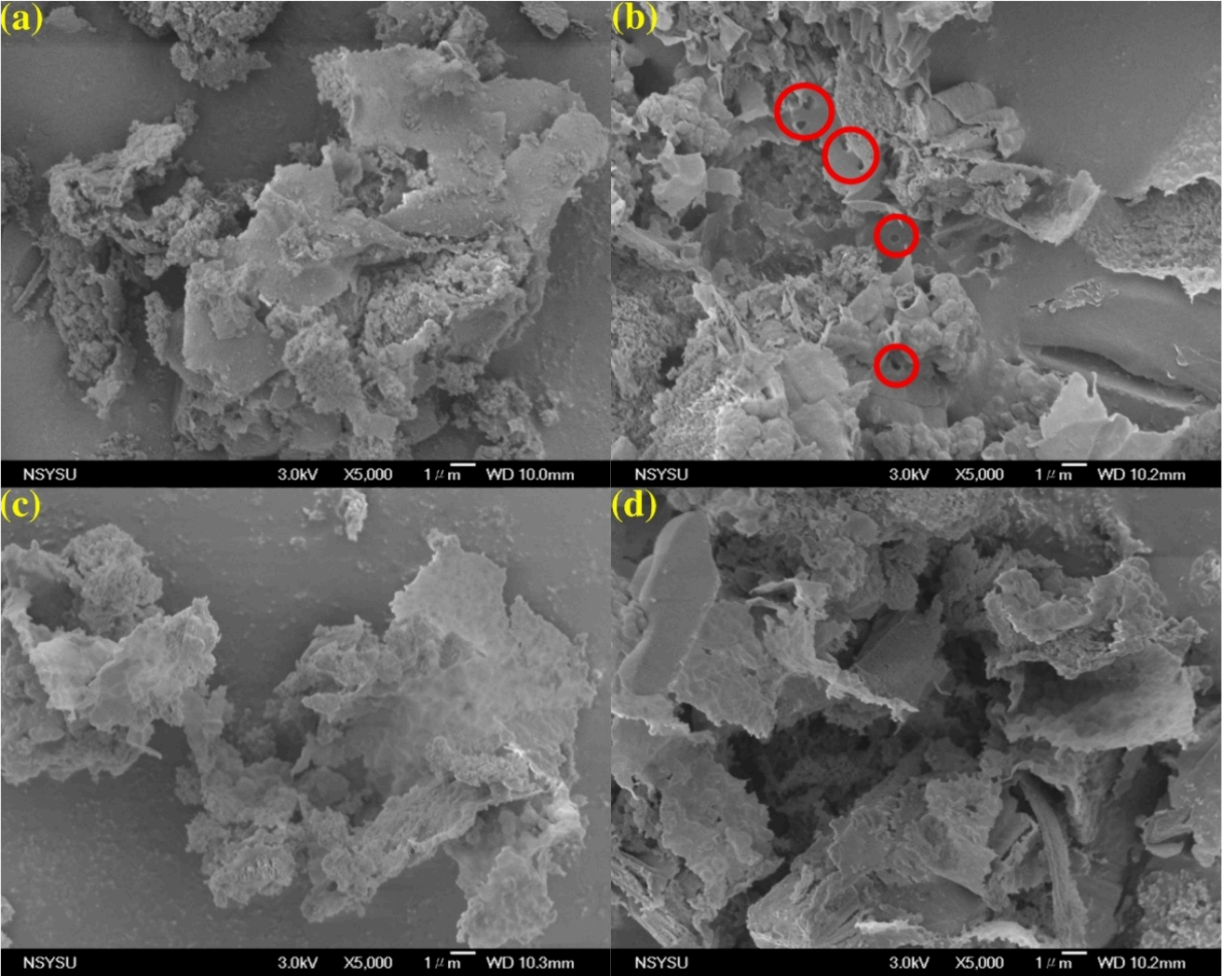
SEM images of (a) CN, (b) 0.1 PCN, (c) 0.2 PCN, and (d) 0.5 PCN samples at a magnification of ×5k.

XRD analysis was employed to examine the crystalline structure of CN and 0.1, 0.2, and 0.5 PCN (Figure 2a). Pristine CN showed two prominent diffraction peaks at 12.87° and 27.86°, corresponding to the (100) and (002) crystal planes of g-C_3_N_4_, respectively. These peaks represent the in-plane structural peaking of tri-s-triazine units (100) and the interlayer stacking of aromatic layers (002).^27,28^ The PCN samples retained these characteristic peaks, indicating the preservation of the basic g-C_3_N_4_ framework. However, with an increasing H_3_PO_4_ concentration, both peaks broadened and decreased in intensity. This suggests a reduction in the planar domain size and potentially an expansion of the interlayer distance, consistent with exfoliation and phosphorus incorporation during the acid treatment.

**Figure 2:**
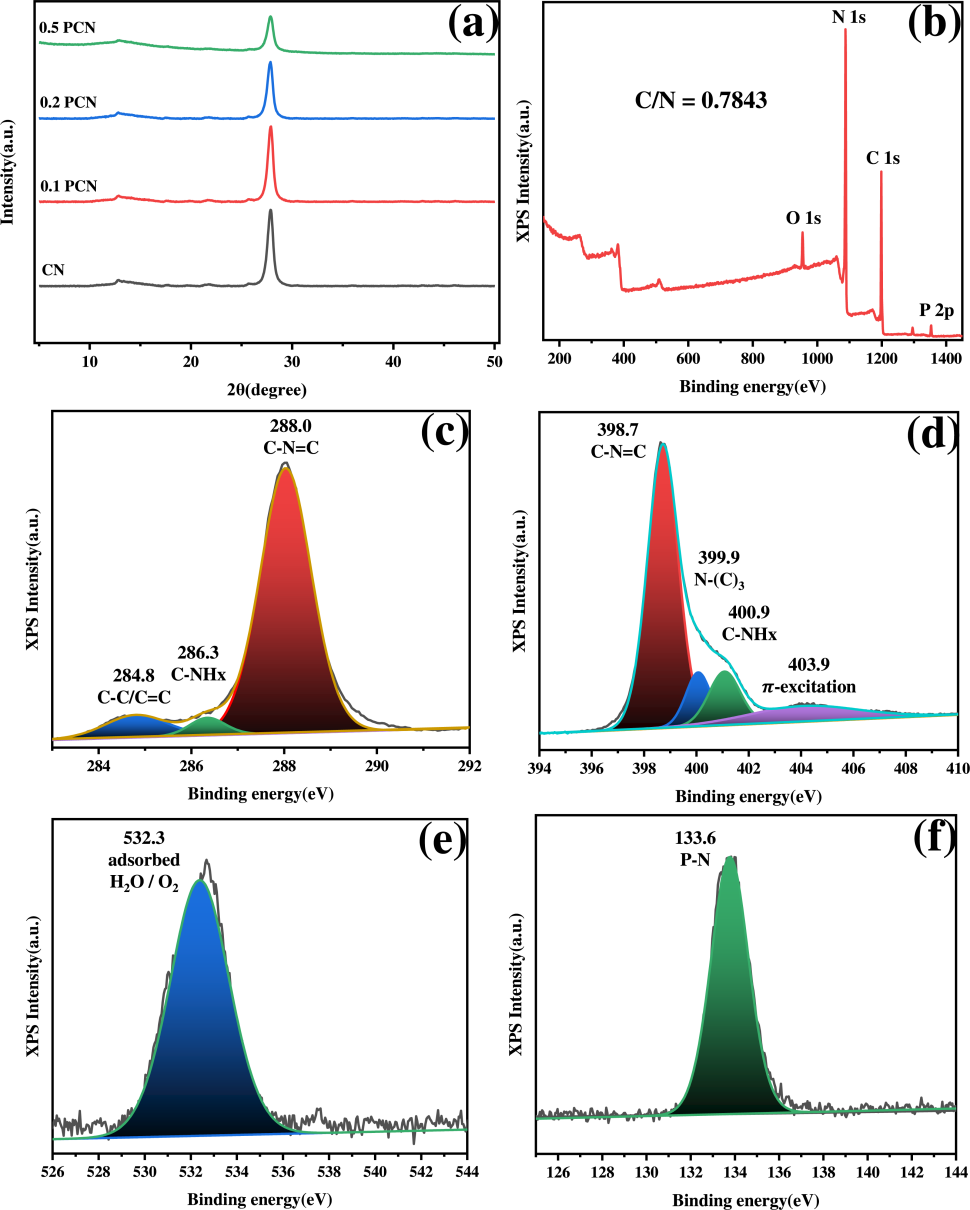
(a) XRD patterns of the CN and PCN samples. High-resolution XPS spectra of (b) survey spectra, (c) C 1s, (d) N 1s, (e) O 1s, and (f) P 2p of 0.1 PCN.

XPS was used to analyze the surface elemental composition and chemical states of the 0.1 PCN sample (Figure 2b). The survey spectrum confirmed that 0.1 PCN consisted mainly of C and N, with small amounts of P and O detected, similar to pristine CN (Figure S1). High-resolution C 1s and N 1s for CN and PCN samples are detailed in Table S1, with the surface C/N atomic ratios. Elemental analysis (Table S2) revealed a C/N atomic ratio of approximately 0.7843 for 0.1 PCN, agreeing well with the theoretical value of 0.75 for g-C_3_N_4_. The slightly higher C/N ratio observed for CN compared to 0.1 PCN might be due to the difference in NH_3_ evolution during polymerization. The high-resolution C 1s spectrum (Figure 2c) was deconvoluted into three peaks at 284.8 eV (adventitious C–C/C=C), 286.3 eV (C-NH_*x*_), and 288.0 eV (C–N=C in triazine rings). The N 1s spectrum (Figure 2d) showed four peaks at 398.7 eV (C–N=C), 399.9 eV (N-(C)_3_), 400.9 eV (C-NH_*x*_), and 403.9 eV (π-excitation bonds).^29^ The O 1s peak at 532.3 eV (Figure 2e) is attributed to surface-adsorbed H_2_O or O_2_.^30^ Importantly, the P 2p XPS spectrum of 0.1 PCN (Figure 2f) exhibits a peak at 133.6 eV, characteristic of P–N bonding, confirming successful phosphorus incorporation from H_3_PO_4_ treatment.^31^ Studies have shown that nonmetallic doping in g-C_3_N_4_, such as with phosphorus, introduces localized electronic defects. These defect states are formed near the conduction band edge, effectively altering the band structure and acting as electron traps. This process promotes charge separation and enhances the photocatalytic performance. Our experimental XPS data, particularly the change in binding energies, align well with these theoretical findings, collectively demonstrating that phosphorus doping effectively modulates the electronic structure of g-C_3_N_4_.^32–35^

### 3.2 Optical and Photoelectric Properties

The optical absorption properties were investigated by using UV–vis diffuse reflectance spectroscopy (DRS). As shown in Figure 3a, compared to CN, the PCN samples exhibited enhanced visible-light absorption, with their absorption edges red-shifted (bathochromic shift). The redshift intensified with H_3_PO_4_ concentration up to 0.1 M, reaching an absorption edge of approximately 630 nm for 0.1 PCN, before slightly blue-shifting at higher concentrations (0.2 PCN, 0.5 PCN). This modulation was also reflected in the samples’ color variations. All materials showed characteristic absorption bands below 400 nm (π–π* transitions in tri-s-triazine units) and extending into the visible region (n−π* transitions). The optical band gap energies (*E*_g_) were estimated using Tauc plots derived from the Kubelka–Munk function in eq 2:

$$\alpha h\nu =A(h\nu -E_{g}{)}^{r}$$ 2

where α is the absorption coefficient, hν is the photon energy, *A* is the constant, and *n* = 2 for direct band gap semiconductors like g-C_3_N_4_. The calculated band gaps (*E*_g_) were 2.95, 2.85, 2.94, and 2.99 eV for CN, 0.1 PCN, 0.2 PCN, and 0.5 PCN, respectively (Figure 3b). The H_3_PO_4_ treatment thus modifies both the band structure and morphology.

**Figure 3:**
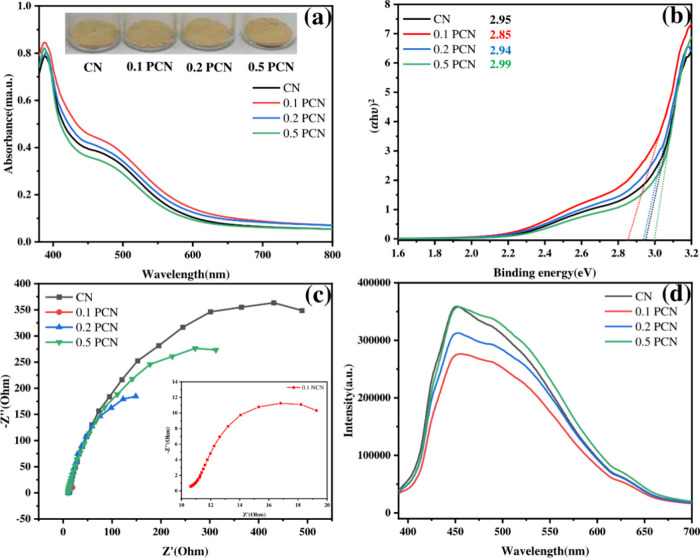
(a) DRS, (b) Tauc plot, (c) EIS, and (d) PL of the CN and PCN samples.

Electrochemical impedance spectroscopy (EIS) and photoluminescence (PL) spectroscopy were used to probe the charge carrier separation and transport efficiency. The smaller semicircle diameter in the Nyquist plot for 0.1 PCN compared to CN (Figure 3c) indicates lower charge transfer resistance at the electrode/electrolyte interface, suggesting more efficient interfacial charge transfer in 0.1 PCN. Furthermore, the PL emission intensity of 0.1 PCN (excited at 380 nm, emission peak ∼ 455 nm) was significantly lower than that of pristine CN (Figure 3d), indicating that the recombination of photogenerated electron–hole pairs was effectively suppressed in the 0.1 PCN sample.

In comparison, a study on B-doped g-C_3_N_4_ (BCN 1:0.15) reported a band gap of 2.65 eV and a red-shifted absorption edge to 507 nm. Similarly, S-doped g-C_3_N_4_ (BSCN) was found to have a band gap of 2.9 eV and an absorption edge around 480 nm. This demonstrates that our material, with its absorption edge red-shifted to 630 nm, achieves a broader photoresponse range in the visible spectrum.^32,36^

Furthermore, our analysis provides direct evidence of enhanced charge carrier separation. The significantly lower PL intensity of 0.1 PCN compared with pristine g-C_3_N_4_ confirms the effective suppression of electron–hole recombination. The smaller diameter of the semicircle in the EIS Nyquist plot further indicates lower charge transfer resistance and higher transfer efficiency. While the literature confirms that other dopants like sulfur or boron can also improve charge separation by modifying the electronic structure and creating defect sites,^32,36^ the extent of enhancement achieved by phosphorus doping in our material is demonstrably significant.

### 3.3 Photocatalytic Degradation of TMP

The photocatalytic performance of the synthesized materials was evaluated by monitoring the degradation of TMP under visible-light irradiation (6 W 405 nm LED). Figure 4a shows the degradation profiles over time (TMP calibration curve is shown in Figure S2). The 0.1 PCN photocatalyst demonstrated the highest activity, achieving >99.00% TMP removal within 90 min, significantly outperforming CN and the other PCN samples. For comparison, irradiation with a 300 W Xe lamp (likely full spectrum, unless filtered) resulted in only 78.48% degradation after 3 h (Figure 4b). While degradation occurred under 6W 365 nm LED light, the efficiency was considerably higher under 6 W 405 nm LED irradiation, confirming it as the optimal light source for this system.

**Figure 4:**
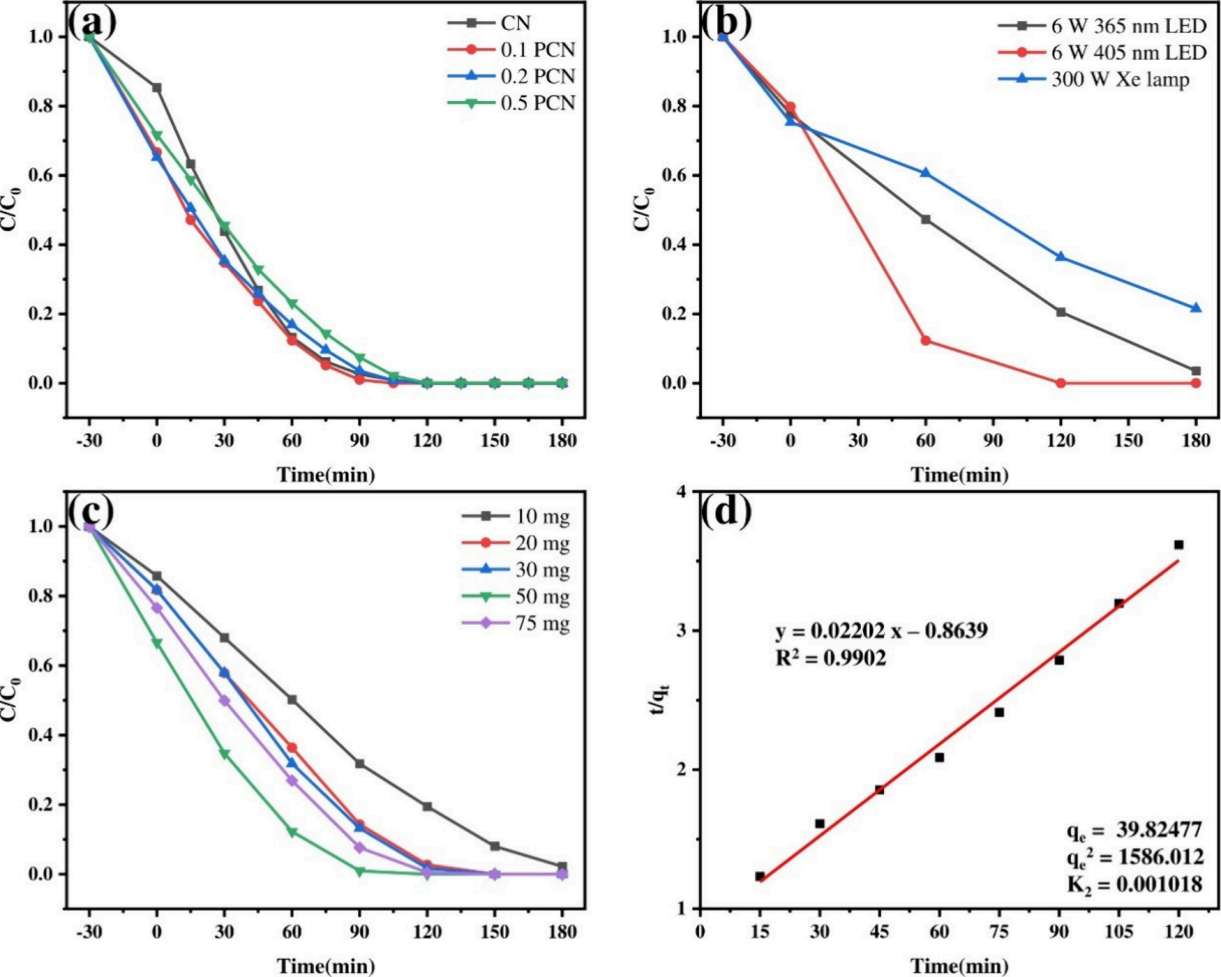
(a) Photocatalytic degradation of TMP on CN and PCN samples under visible-light irradiation. The effects of (b) different light sources, (c) different catalyst dosages, and (d) pseudo-second-order kinetics of degradation efficiency on TMP photodegradation by 0.1 PCN.

The effect of catalyst loading on TMP degradation was investigated using quantities from 10 to 75 mg (Figure 4c). Degradation efficiencies after 90 min increased from 68.22% (10 mg) to a maximum of 99.02% (50 mg), attributed to the increased number of available active sites. However, increasing the loading further to 75 mg decreased the efficiency to 92.36%, likely due to reduced light penetration and increased scattering effects. Therefore, 50 mg was determined to be the optimal catalyst loading for subsequent experiments.

To analyze the degradation kinetics, pseudo-first-order and pseudo-second-order models were applied to the experimental data (Figure S3 and Figure 4d). The pseudo-second-order model (*R* ^2^ = 0.9902) provided a significantly better fit than the pseudo-first-order model (*R* ^2^ = 0.9733). This indicates that the photocatalytic degradation of TMP over 0.1 PCN primarily follows pseudo-second-order reaction kinetics under these conditions.

A key aspect of our work is the challenging reaction conditions under which we achieved a high efficiency. Our study utilized an initial TMP concentration of 100 μM (29.03 mg/L), which is notably higher than the concentrations typically reported in comparable literature (e.g., a related study on Ag/h-MoO_3_ degradation used 10 mg/L).^4^ Despite the high initial concentration and a low-power 6 W 405 nm LED light source, our optimized catalyst achieved over 99% TMP degradation in 90 min. This exceptional performance under strenuous conditions highlights the superior efficiency and robustness of our material. We contend that this high degradation efficiency, even with a challenging pollutant load, serves as a strong indicator of our catalyst’s potential for real-world applications.

### 3.4 Reaction Mechanism

To identify the primary reactive species responsible for TMP degradation, scavenger experiments were performed. As shown in Figure 5a,b, the addition of scavengers for hydroxyl radicals (·OH; IPA), electrons (e^–^; NaNO_3_), holes (h^+^; AO), singlet oxygen (^1^O_2_; l-His), and superoxide radicals (·O_2_^–^; p-BQ) significantly impacted the degradation efficiency over 0.1 PCN. After 90 min, degradation efficiencies were inhibited to varying degrees in the presence of scavengers for e^–^ (79.37%), ^1^O_2_ (35.60%), h^+^ (32.00%), and ·O_2_^–^ (14.92%), while the ·OH scavenger (IPA) had minimal effect (efficiency remained high, suggesting that ·OH is not dominant). The substantial inhibition observed upon adding p-BQ, l-His, and AO indicates that ·O_2_^–^, ^1^O_2_, and h^+^ are the key reactive species driving TMP degradation in this system. The relative importance appears to follow the order: ·O_2_^–^ > h^+^ > ^1^O_2_ > e^–^ > ·OH.

**Figure 5:**
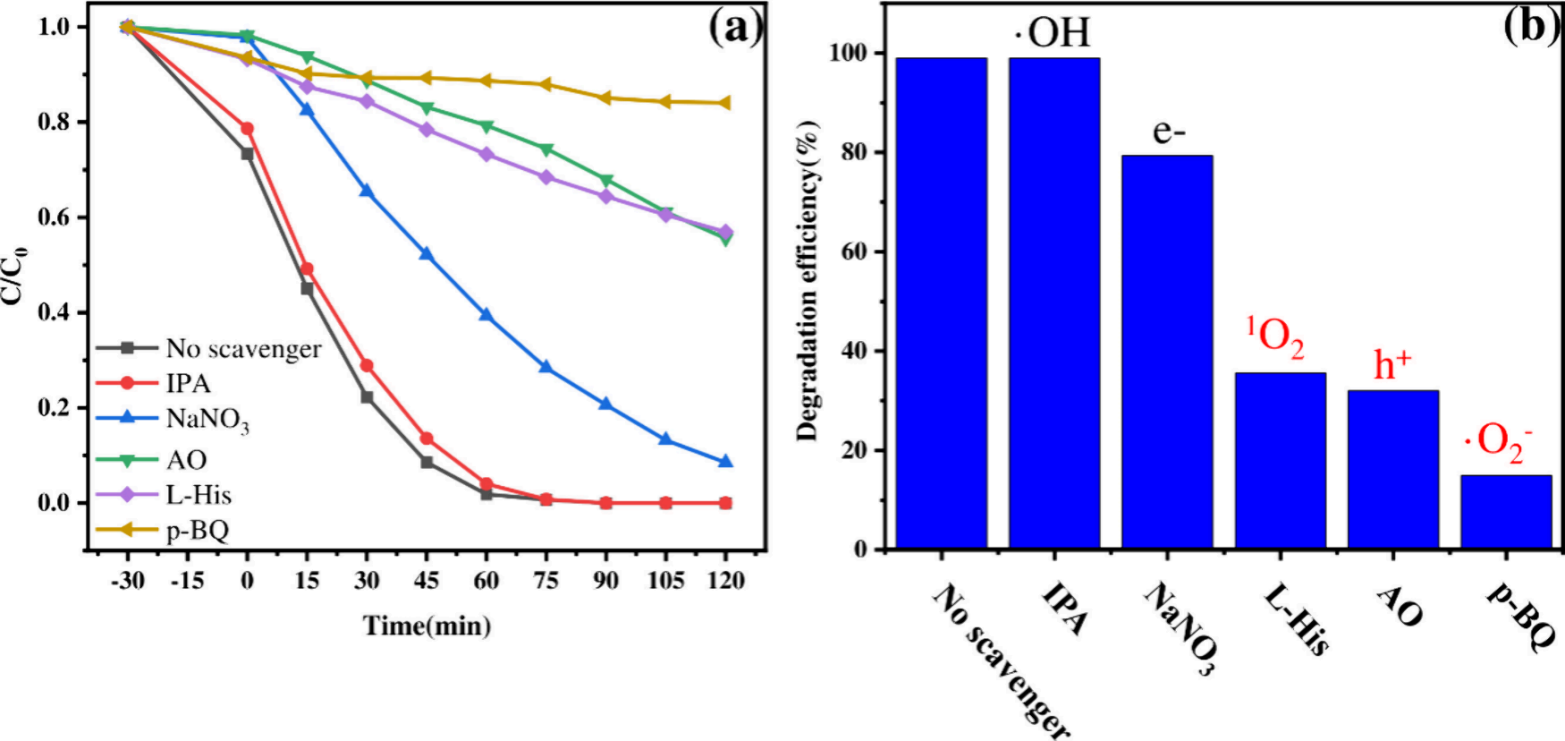
(a) Photocatalytic degradation of TMP over 0.1 PCN by quenching photolysis-induced reactive species. (b) Degradation efficiency of TMP over 0.1 PCN by quenching photolysis-induced reactive species. The initial concentrations of IPA, NaNO_3_, AO, l-His, and p-BQ were 0.1 mol L^–1^.

Beyond the identified reactive species, the catalyst’s surface characteristics also play a crucial role. The observed high selectivity for cationic dyes strongly suggests that the catalyst surface is negatively charged. We attribute this negative charge to the incorporation of phosphorus from the H_3_PO_4_ treatment, which facilitates electrostatic attraction with positively charged pollutants. This combination of enhanced surface area from exfoliation, as indicated by SEM images, and favorable electrostatic interactions explains the material’s excellent performance.

### 3.5 Reaction Intermediates and Degradation Pathway

Based on LC-MS analyses (Figure S4), potential degradation intermediates of TMP were identified, and a plausible degradation pathway is proposed (Figure 6). The main degradation steps appear to involve hydrogenation, oxidation, and molecular cleavage reactions. Initial steps may involve hydrogenation, leading to intermediates P1, P2, and P3.^37^ Fragmentation of P1 could yield P5, P6, and P7, while P2 cleavage could form P8.^37–39^ Oxidation of P2/P3 might form P9.^37^ Further oxidative cleavage of P4’s product could generate P10/P11,^37,38^ followed by oxidation of P11 to form P12.^37^

**Figure 6:**
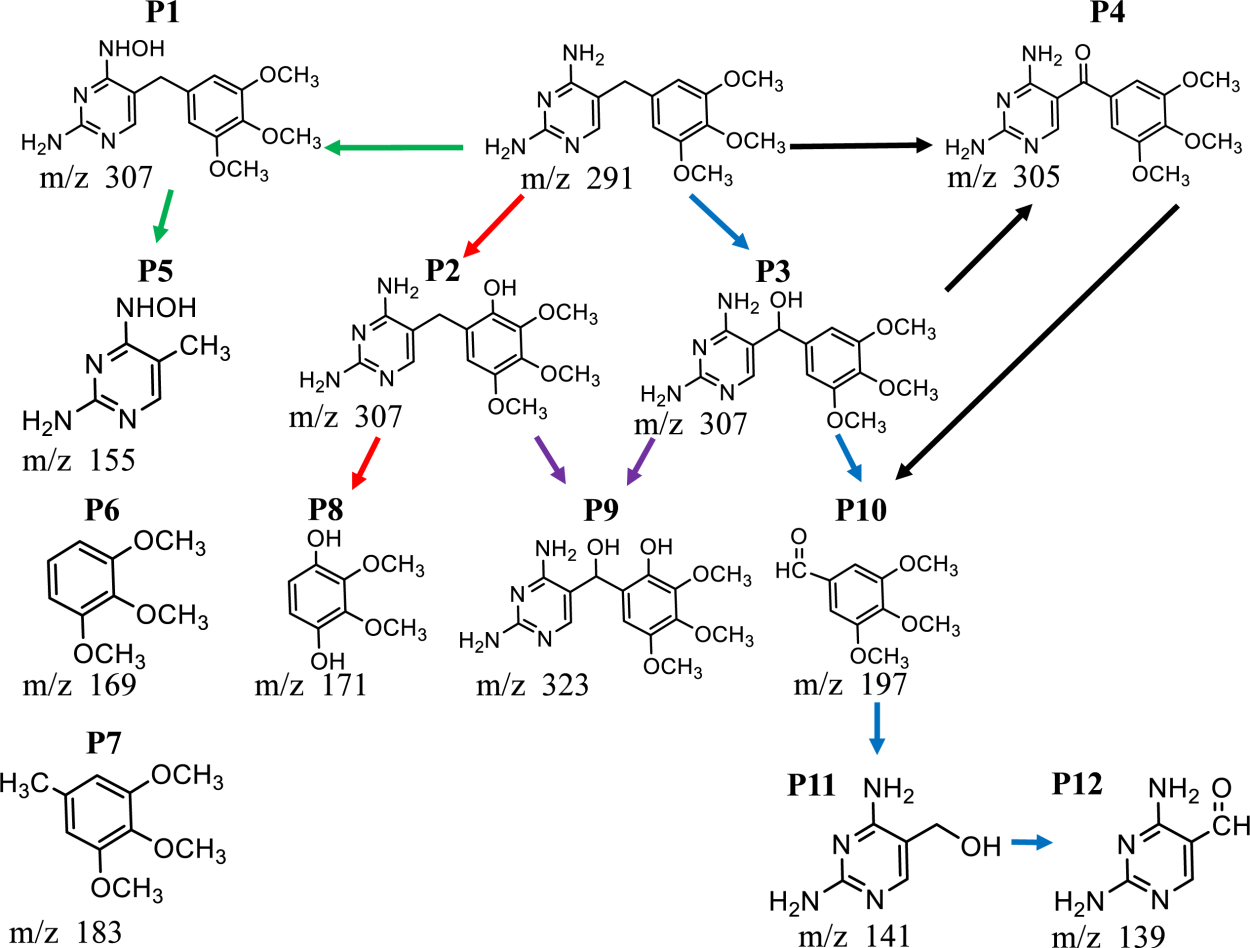
Possible degradation pathways of TMP.

To assess the potential environmental risks of TMP and its degradation intermediates, we evaluated their ecotoxicity using a quantitative structure–activity relationship (QSAR) method with the Toxicity Estimation Software Tool (T.E.S.T.).^4^ The assessment, as presented in Table S3, included an analysis of acute toxicity, developmental toxicity, and bioaccumulation factors for the identified intermediates.

### 3.6 Stability and Practical Application Evaluation

The stability and recyclability of the optimal 0.1 PCN catalyst were assessed through four consecutive photocatalytic cycles. After each 90 min cycle, the catalyst was recovered, washed with DI water and methanol, dried, and reused with a fresh TMP solution. As shown in Figure 7a, the degradation efficiency decreased only slightly, by 5.23%, after the fourth cycle, demonstrating good operational stability. XRD analysis confirmed that the catalyst morphology remained largely unchanged after cycling (Figure 7b). Furthermore, the minimal 5.23% decrease in degradation efficiency after four cycles indicates that serious structural decay or leaching did not occur within this time frame, further supporting its structural robustness.

**Figure 7:**
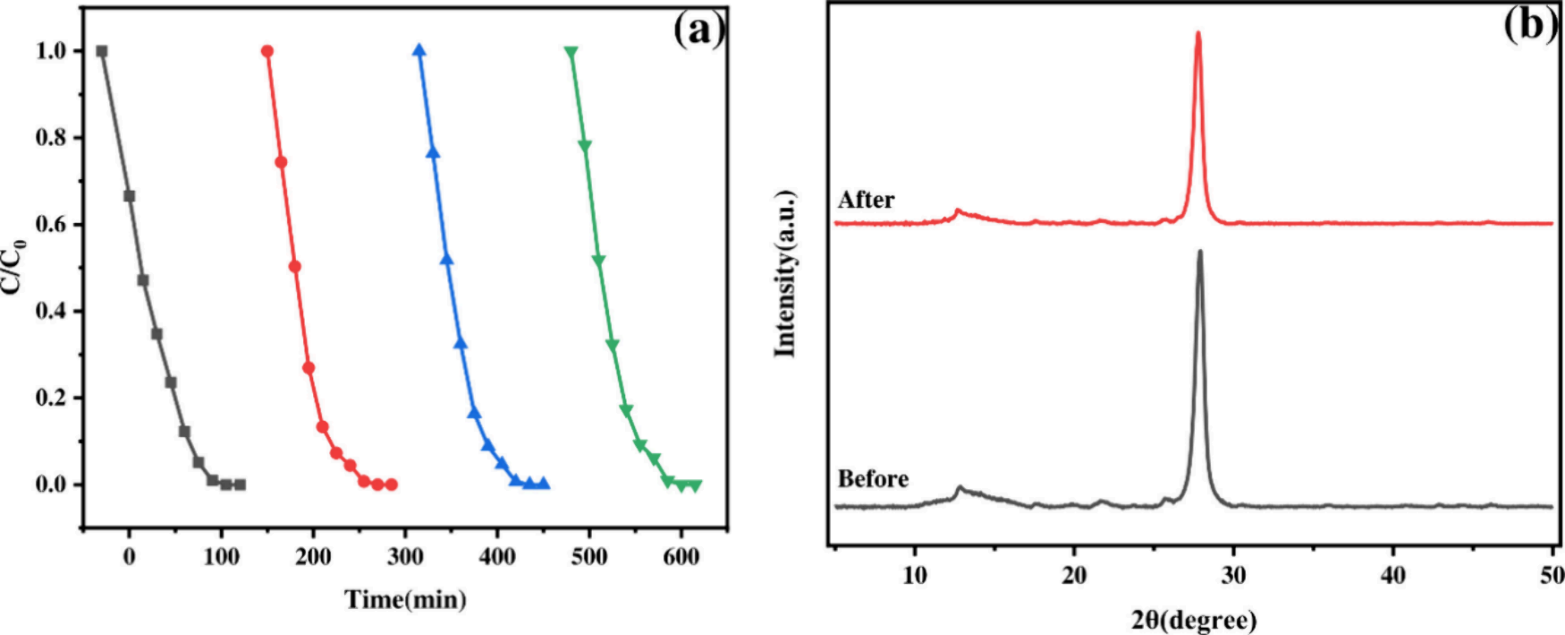
(a) Cycling test of 0.1 PCN for photocatalytic degradation of TMP under visible-light irradiation. (b) XRD patterns were fresh and used 0.1 PCN.

To evaluate performance under more realistic conditions, degradation experiments were conducted in municipal tap water and natural lake water samples, which were pretreated by centrifugation and membrane filtration to remove suspended solids (Figure S5). The degradation efficiency of TMP was only marginally affected by these complex water matrices compared to DI water, indicating the catalyst’s strong resistance to interference from common inorganic salts and organic matter.

The photocatalyst’s selectivity, potentially linked to the dominant role of ^1^O_2_ and ·O_2_^–^, was probed using various organic pollutants (Figure S6). Cationic dyes (MB, RB, and CV) were efficiently degraded, likely due to favorable electrostatic attraction to the PCN surface (suggesting a negative surface charge under reaction conditions), while the anionic dye MO showed negligible degradation (Figure S6a). Among the tested antibiotics (Figure S6b), TMP was degraded most effectively (>99% in 90 min). Tetracyclines (TC, CTC, OTC, DC) showed moderate degradation (40–66%), while sulfonamides (SMZ, SMX) exhibited lower degradation rates (24–28%). These results highlight a structure-dependent selectivity in the degradation process.

Finally, the performance of 0.1 PCN was benchmarked against commercial P25 TiO_2_ and ZnO under identical 6W 405 nm LED irradiation (Figure 8a,b). 0.1 PCN showed vastly superior TMP degradation (>99% in 90 min) compared to P25 (16.14%) and ZnO (11.61%). Similar superiority was observed for RB degradation (Figure S7a), where 0.1 PCN also exhibited a higher initial adsorption capacity. Visual confirmation of RB decolorization is provided in Figure S7b. Comparison with literature data for similar systems (Table S4) further underscores the high photocatalytic performance of the synthesized 0.1 PCN material.

**Figure 8:**
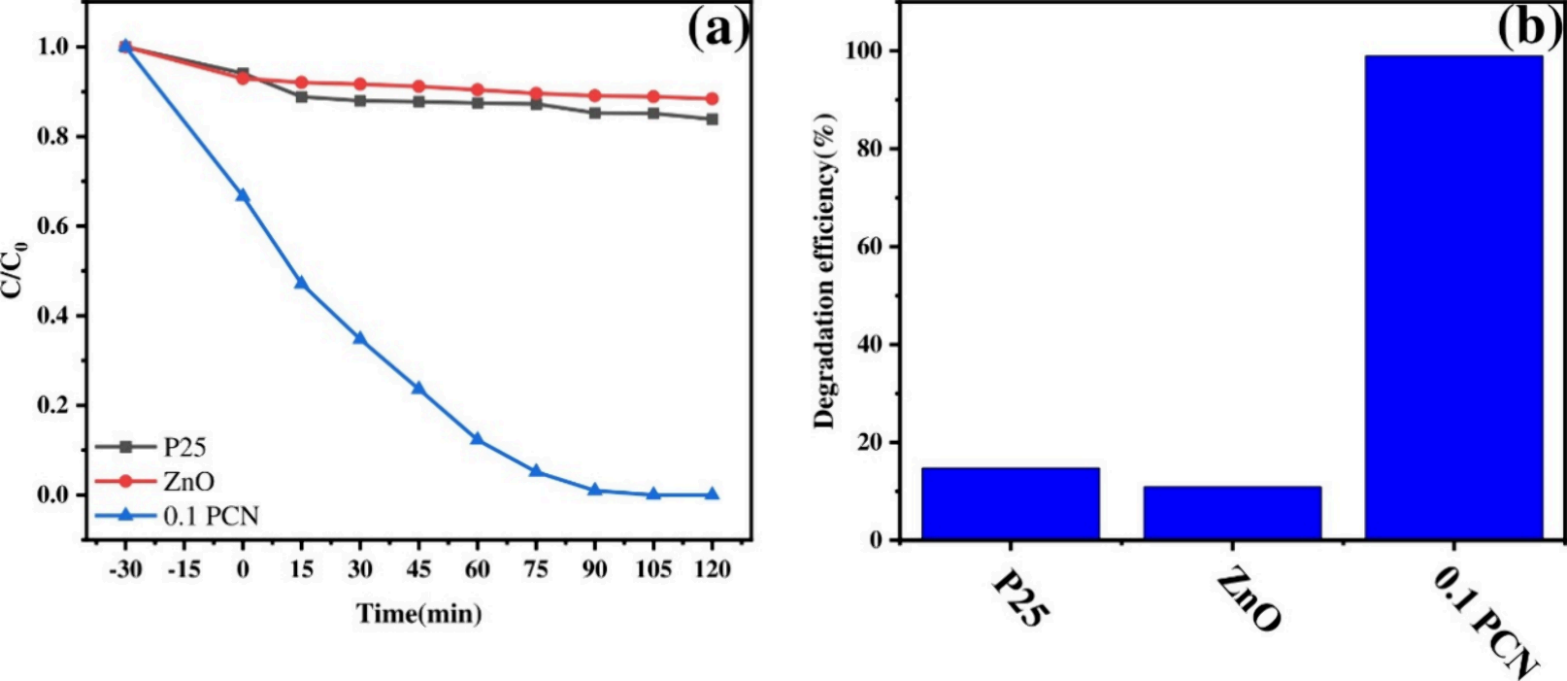
(a) Compared with the degradation of TMP in commercial P25 and ZnO under a 6 W 405 nm LED. (b) Degradation efficiency of TMP in commercial P25 and ZnO.

## 4 Conclusions

In summary, phosphorus-doped graphitic carbon nitride (P-doped g-C_3_N_4_, PCN) photocatalysts were successfully synthesized via thermal polymerization, and the efficiency for degrading the antibiotic trimethoprim (TMP) under visible light was systematically investigated. The optimized material, 0.1 PCN (prepared using 0.1 M H_3_PO_4_), demonstrated excellent photocatalytic performance, achieving >99% degradation of TMP (100 μM) within 90 min under 6 W 405 nm LED irradiation. The enhanced activity was attributed to the beneficial effects of phosphorus doping and H_3_PO_4_-induced exfoliation, which improved the visible-light absorption and promoted efficient charge carrier separation. Mechanistic investigations confirmed that singlet oxygen (^1^O_2_) and superoxide radicals (·O_2_^–^) were the dominant reactive oxygen species responsible for TMP degradation. The 0.1 PCN catalyst also exhibited high stability and effective recyclability over multiple cycles and maintained robust performance in real water samples (tap and lake water). This work demonstrates that P-doped g-C_3_N_4_ is a promising, effective, and sustainable metal-free photocatalyst for removing antibiotic contaminants from water.

## Supplementary Material



## Notes

Figure S1: High-resolution XPS spectra of (a) survey spectra, (b) C 1s, (c) N 1s and (d) O 1s of CN; Figure S2: (a) HPLC chromatogram and (b) calibration curve of TMP concentration; Figure S3: plots of −ln­(*C*/*C*_0_) against t based on the pseudo-first-order kinetic model for degradation of TMP on 0.1 PCN; Figure S4: results of LC-MS that the reaction intermediates of TMP degradation under different degradation times (a) BK, (b) adsorption 30 min, (c) 15 min, (d) 30 min, (e) 45 min, (f) 60 min, (g) 75 min, and (h) 90 min; Figure S5: photocatalytic degradation of TMP in DI water, tap water, and lake water by 0.1 PCN (the original pH values were pH 6.87 at 26 °C for tap water and pH 8.98 at 28 °C for lake water); Figure S6: photocatalytic performance of 0.1 PCN for removal of various pollutants, including (a) methylene blue (10 μM), rhodamine B (10 μM), crystal violet (10 μM), methyl orange (30 μM), (b) trimethoprim (100 μM), sulfamethazine (100 μM), sulfamethoxazole (100 μM), tetracycline (100 μM), chlortetracycline (100 μM), oxytetracycline (100 μM), and doxycycline (100 μM); Figure S7: (a) compared with the degradation of RB in commercial P25 and ZnO under two 3 W 405 nm LED and (b) illustrative images depicting the degradation of RB using different photocatalysts—commercial P25, ZnO, and 0.1PCN; Table S1: XPS fitting data of CN and 0.1 PCN; Table S2: elemental analysis results of CN and 0.1 PCN; Table S3: acute toxicity, bioaccumulation factor, developmental toxicity, and mutagenicity value of TMP and degradation intermediates; and Table S4: comparisons of TMP degradation catalyzed by 0.1 PCN and previous reported catalysts (PDF)
