# Supplementary material for: Phosphorus-Doped Carbon Nitride Materials for Enhanced Photocatalytic Degradation of Organic Pollutants under Visible-Light Irradiation
Source: ACS Omega. 2025 Sep 8;10(36):41457–66. doi: 10.1021/acsomega.5c04783 (PMC12444591; doi:10.1021/acsomega.5c04783)
Supplement: Supplementary file 1 [file ao5c04783_si_001.pdf]

Supporting Information

Phosphorus-Doped Carbon Nitride Materials for Enhanced Photocatalytic Degradation of Organic Pollutants under Visible Light Irradiation

*Yi-Zhen Huang, Yu-Shen Lin, Yu-Shan Lin, Tai-Chia Chiu, Cho-Chun Hu**

Department of Applied Science, National Taitung University, No. 369, Sec. 2, University Road, Taitung City, Taitung County 95092, Taiwan (R.O.C.)

Corresponding Author

Department of Applied Science, National Taitung University, No. 369, Sec. 2, University Road, Taitung City, Taitung County 95092, Taiwan (R.O.C.)

Tel.: +886 (089)517991#6434. E-mail address: cchu@nttu.edu.tw

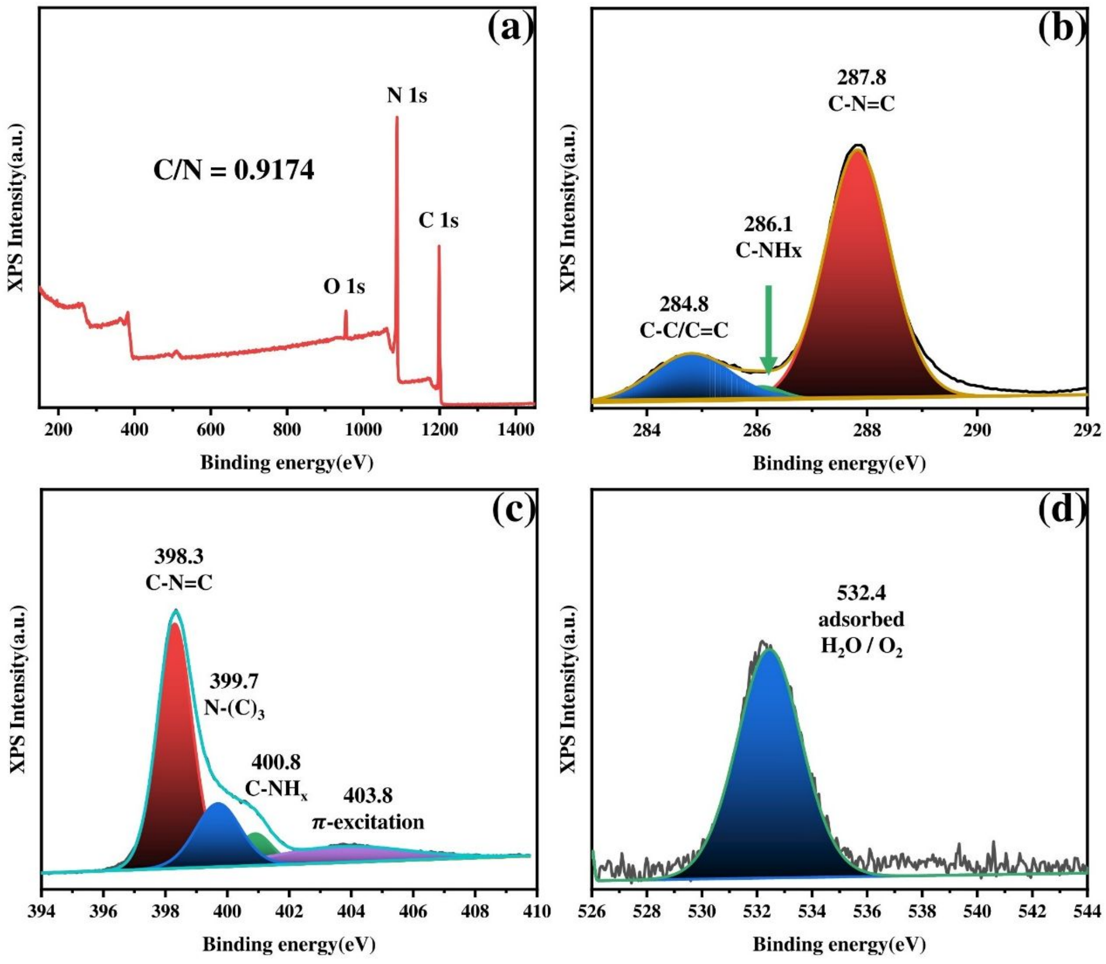

Figure S1. High-resolution XPS spectra of (a) survey spectra, (b) C 1s, (c) N 1s and (d) O 1s of CN.

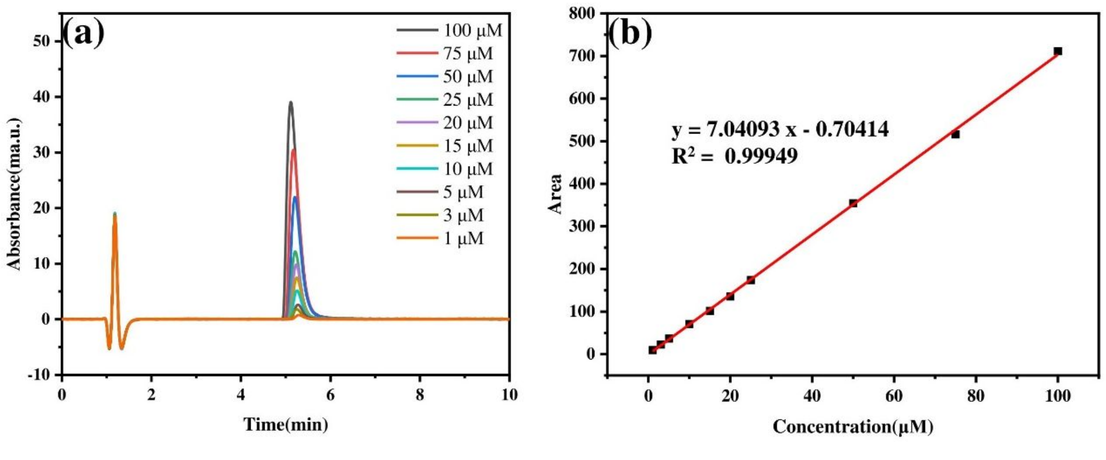

Figure S2. The (a) HPLC chromatogram and (b) calibration curve of TMP concentration.

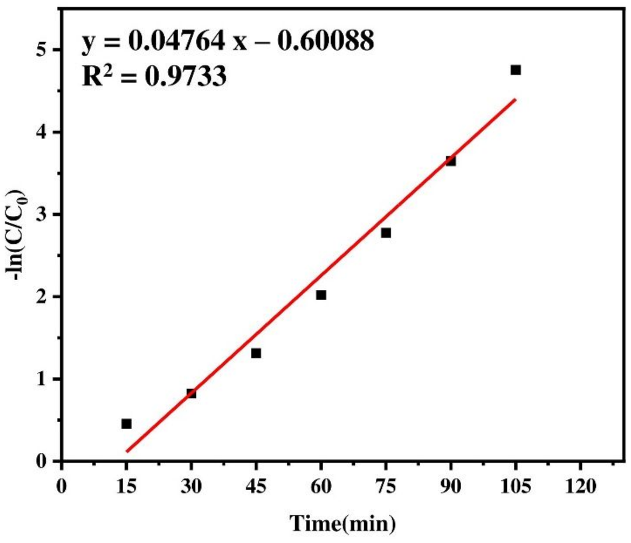

Figure S3. Plots of -ln(C/C_0) against t based on the pseudo-first-order kinetic model for degradation of TMP on 0.1 PCN.

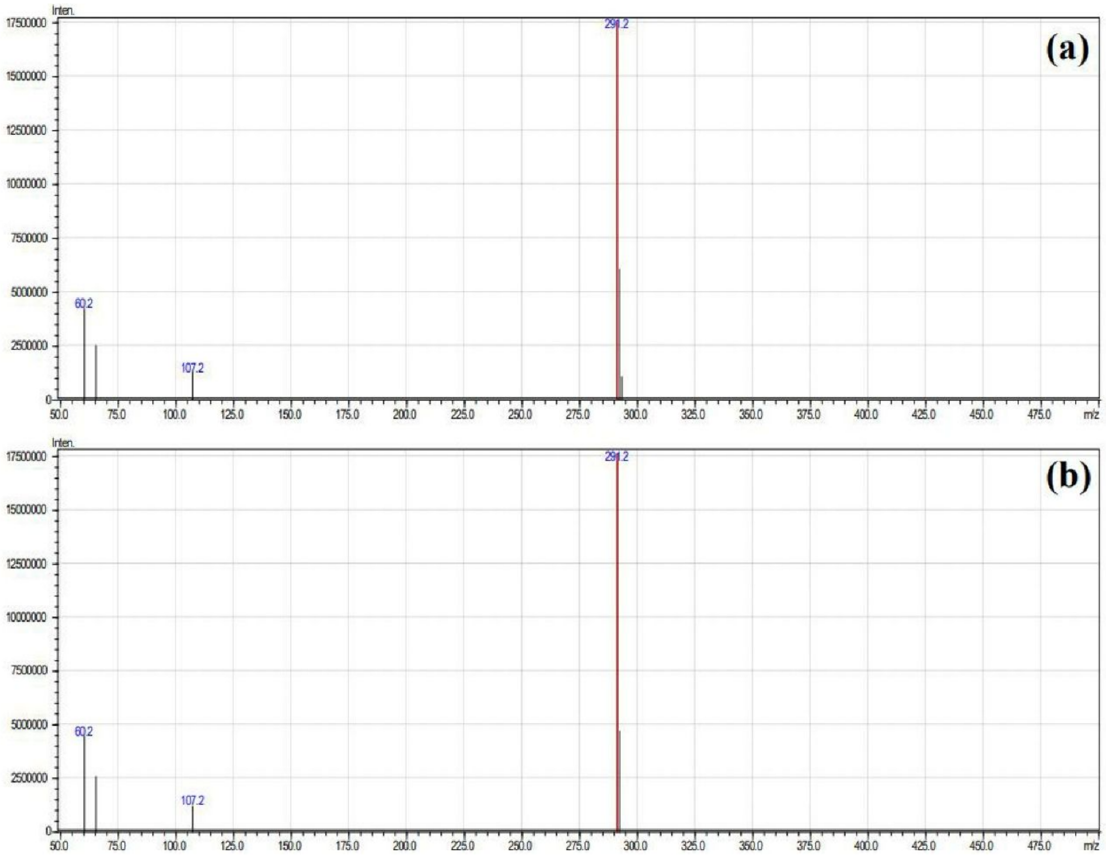

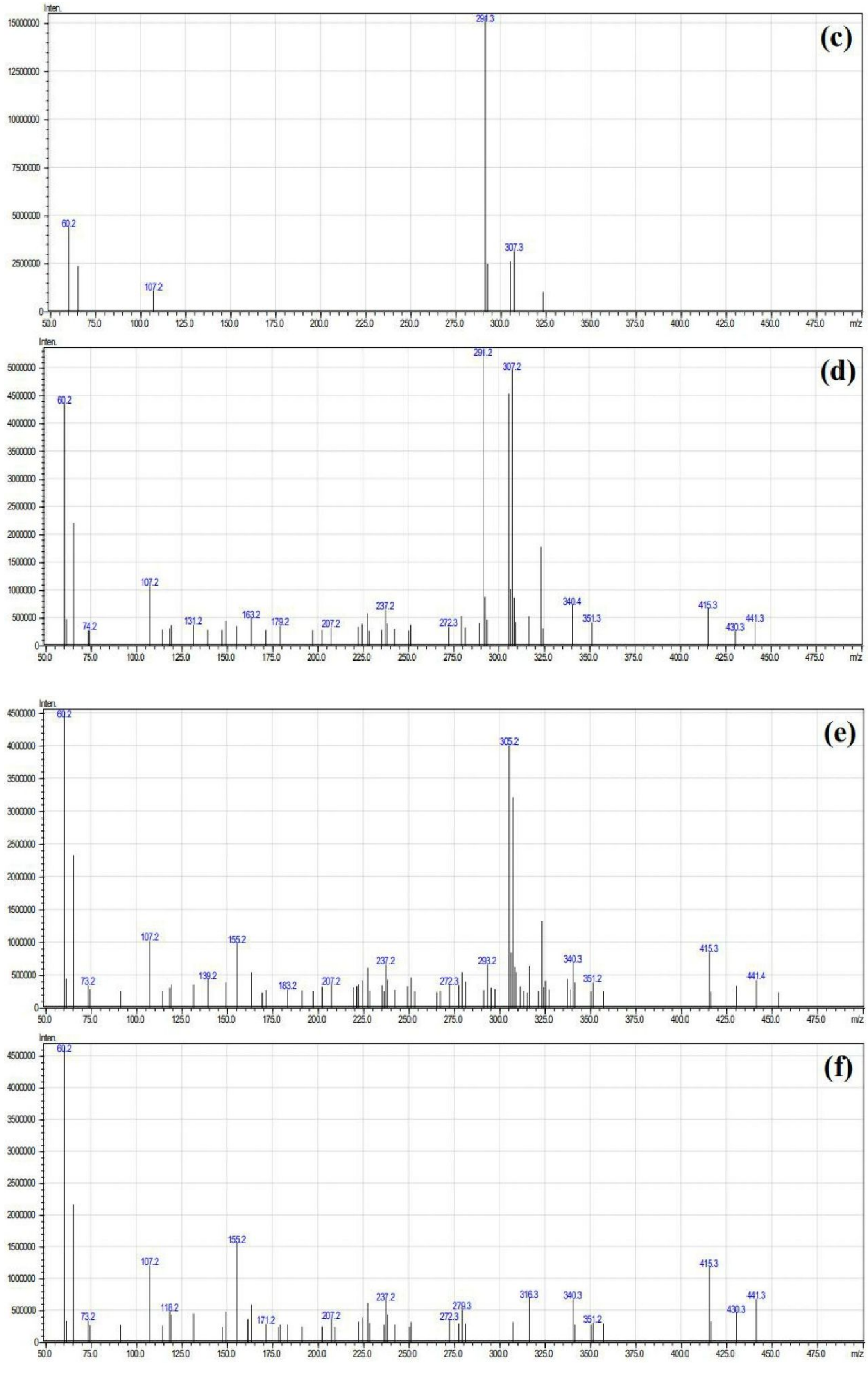
(c)
Inten.
291.3
307.3
60.2
107.2
m/z
(d)
Inten.
291.2
307.2
60.2
74.2
107.2
131.2
163.2
179.2
207.2
237.2
272.3
340.4
351.3
415.3
430.3
441.3
m/z
(e)
Inten.
60.2
305.2
73.2
107.2
139.2
155.2
183.2
207.2
237.2
272.3
293.2
340.3
351.2
415.3
441.4
m/z
(f)
Inten.
60.2
73.2
107.2
118.2
155.2
171.2
207.2
237.2
272.3
279.3
316.3
340.3
351.2
415.3
430.3
441.3
m/z

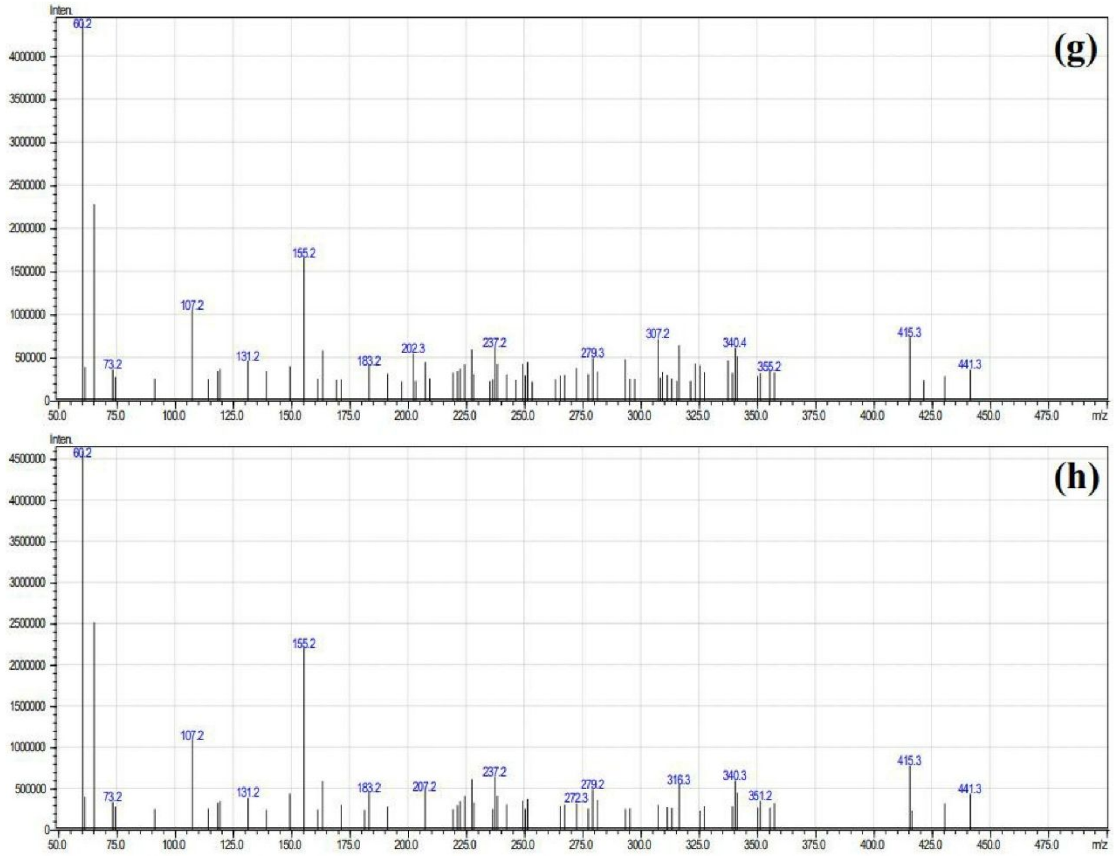

Figure S4. The results of LCMS that the reaction intermediates of TMP degradation under different degradation time (a) BK, (b) adsorption 30 min, (c) 15 min, (d) 30 min, (e) 45 min, (f) 60 min, (g) 75 min, (h) 90 min.

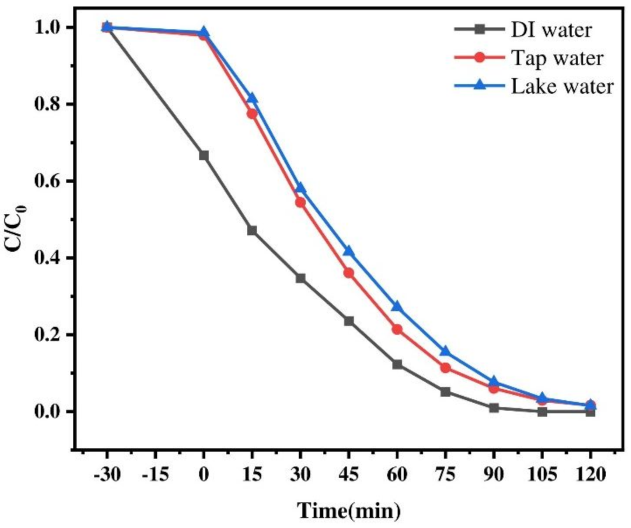

Figure S5. Photocatalytic degradation of TMP in DI water, tap water, and lake water by 0.1 PCN. (The original pH values were pH 6.87 at 26 ˚C for tap water and pH 8.98 at 28 ˚C for lake water.)

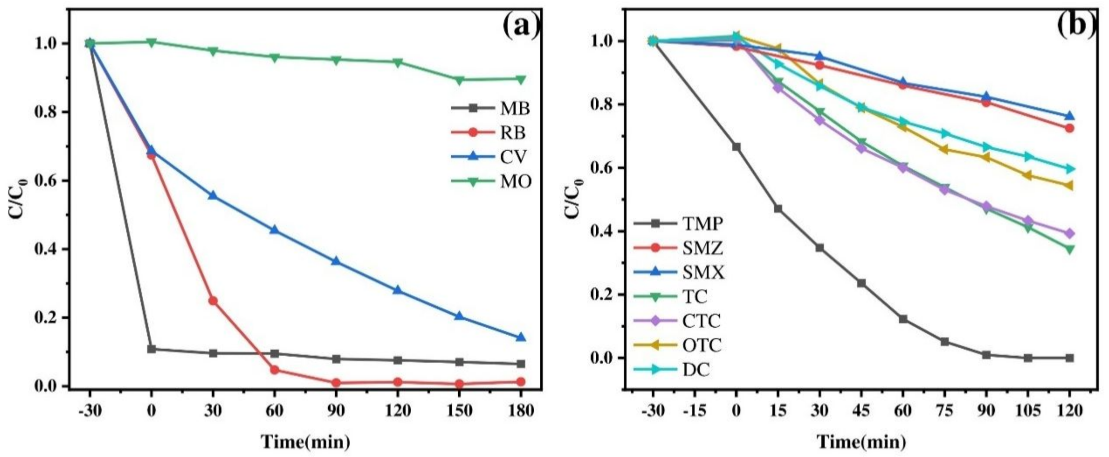

Figure S6. Photocatalytic performance of 0.1 PCN for removal of various pollutants, including (a) methylene blue (10 μM), rhodamine B (10 μM), crystal violet (10 μM), methyl orange (30 μM), (b) trimethoprim (100 μM), sulfamethazine (100 μM), sulfamethoxazole (100 μM), tetracycline (100 μM), chlortetracycline (100 μM), oxytetracycline (100 μM), and doxycycline (100 μM).

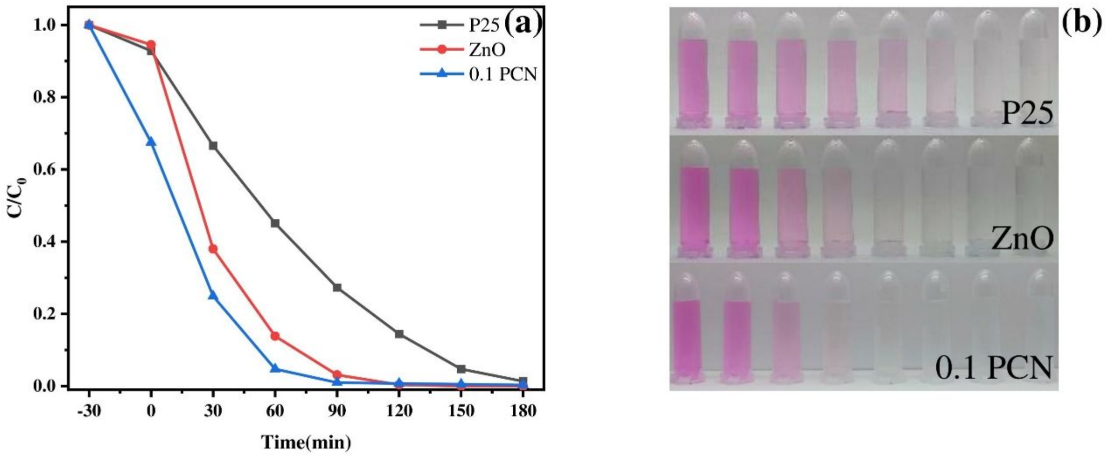

Figure S7. (a) Compared with the degradation of RB in commercial P25 and ZnO under two 3 W 405 nm LEDs. (b) Illustrative images depicting the degradation of RB using different photocatalysts — commercial P25, ZnO, and 0.1PCN.

Table S1. XPS fitting data of CN and 0.1 PCN.

Sample		CN			0.1 PCN		
		Position	Area	%	Position	Area	%
C 1s	C-C/C=C	284.8000	4953.1	18.55%	284.8000	2847.9	9.53%
	$C-NH_x$	286.0883	677.2	2.54%	286.3304	1250.4	4.18%
	C-N=C	287.8183	21076.1	78.92%	288.0251	25793.3	86.29%
N 1s	C-N=C	398.2769	27050.6	56.19%	398.5294	47083.4	64.28%
	$N-(C)_3$	399.7043	9410.5	19.55%	399.9050	7908.4	10.80%
	$C-NH_x$	400.8835	3670.8	7.63%	400.9241	8712.5	11.89%
	π-excitation	403.7555	8006.2	16.63%	403.9020	9544.0	13.03%
O 1s	H-O-H/ O=O	532.4257	6414.3	100.00%	532.3023	9516.7	100.00%
P 2p	P-N	-	-	-	133.5846	2302.8	100.00%

Table S2. The elemental analysis results of CN and 0.1 PCN.

		CN		0.1 PCN	
	Position	Area	%	Area	%
C 1s	288.08	226138.0	46.31%	242438.2	40.86%
N 1s	398.08	443677.6	50.48%	556365.4	52.10%
O 1s	532.08	46006.3	3.22%	74468.0	4.28%
P 2p	133.08	-	-	19428.5	2.75%
C/N	-	0.9174		0.7843	

Table S3. Acute toxicity, bioaccumulation factor, developmental toxicity, and mutagenicity value of TMP and degradation intermediates.

Molecule	Acute Toxicity* LC50 (mg/L)	Bioaccumulation factor	Developmental toxicity**	Mutagenicity***
TMP	10.68	13.83	0.84	0.42
P1	N/A	5.84	0.89	0.63
P2	8.50	8.12	0.91	0.23
P3	23.04	5.99	0.94	0.25
P4	18.92	11.97	0.90	0.25
P5	N/A	1.38	0.58	0.39
P6	93.47	23.95	0.65	0.23
P7	61.69	54.51	0.74	0.30
P8	96.41	9.15	0.62	0.25
P9	10.63	1.59	0.93	0.38
P10	48.81	N/A	0.85	0.12
P11	897.60	0.79	0.31	0.11
P12	305.05	N/A	0.30	N/A

*Acute toxicity: LC50 <10 mg/L is Toxic; 10-100 mg/L is Harmful; >100 mg/L is not harmful.[1]

**Developmental toxicity: value >0.5 is developmental toxicant; <0.5 is developmental non-toxicity.

***Mutagenicity: value >0.5 is mutagenicity positive; <0.5 is mutagenicity negative.

Table S4. Comparisons of TMP degradation catalyzed by 0.1 PCN and previous reported catalysts.

Photocatalysts	Catalyst dosage	$[TMP]_0$	Light source	Degradation time	Degradation rate	Ref.
AgBr/h-MoO_3	25 mg	10 mg/L	300 W Xenon lamp	30 min	100%	[1]
Hierarchical CuO/Cu_2O	20 mg	~29.90 mg/L	500 W Xenon lamp	300 min	48%	[2]
ZnO-Ce	500 mg	0.5 mg/L	10:30 ~ 11:30 sun light (37.0909°N, 2.357°W)	45 min	80%	[3]
OCN_v-U_{40}	60 mg	5 mg/L	1500 W Xenon lamp	90 min	98%	[4]
VUV/K-g-C_3N_4	20 mg	100 mg/L	8 W VUV lamp (10-200 nm)	60 min	91%	[5]
Vis/Cl-g-C_3N_4/PDS	50 mg	20 mg/L	300 W Xenon lamp	120 min	98%	[6]
0.1 PCN	50 mg	~29.03 mg/L	6 W 405 nm LED	90 min	99%	This work

Reference

(1) Cai, Z.; Song, Y.; Jin, X.; Wang, C. C.; Ji, H.; Liu, W.; Sun, X. Highly efficient AgBr/h-MoO(3) with charge separation tuning for photocatalytic degradation of trimethoprim: Mechanism insight and toxicity assessment. *Sci Total Environ* **2021**, *781*, 146754. DOI: 10.1016/j.scitotenv.2021.146754.

(2) Sekar, K.; Chuaicham, C.; Balijapalli, U.; Li, W.; Wilson, K.; F. Lee, A.; Sasaki, K. Surfactant- and template-free hydrothermal assembly of Cu_2O visible light photocatalysts for trimethoprim degradation. *Applied Catalysis B: Environmental* **2021**, *284*, 119741. DOI: 10.1016/j.apcatb.2020.119741.

(3) Berruti, I.; Goncalves, N. P. F.; Calza, P.; Paganini, M. C.; Oller, I.; Polo-Lopez, M. I. Natural solar activation of modified zinc oxides with rare earth elements (Ce, Yb) and Fe for the simultaneous disinfection and decontamination of urban wastewater. *Chemosphere* **2022**, *303* (Pt 2), 135017. DOI: 10.1016/j.chemosphere.2022.135017.

(4) Hasija, V.; Singh, P.; Thakur, S.; Stando, K.; Nguyen, V.-H.; Le, Q. V.; Alshehri, S. M.; Ahamad, T.; Wu, K. C. W.; Raizada, P. Oxygen doping facilitated N-vacancies in g-C3N4 regulates electronic band gap structure for trimethoprim and Cr (VI) mitigation: Simulation studies and photocatalytic degradation pathways. *Applied Materials Today* **2022**, *29*, 101676. DOI: 10.1016/j.apmt.2022.101676.

(5) Lu, W.; Wang, A.; Zhang, Y.; Ren, S.; Zhang, Z. Insights into enhanced degradation of antibiotic trimethoprim in water using a novel K doped g-C_3N_4 photocatalyst under vacuum-UV irradiation: Performance and mechanisms. *Chemical Engineering Journal* **2024**, *495*, 153192. DOI: 10.1016/j.cej.2024.153192.

(6) Chen, J.; Hu, J.; Lin, Y.; Liu, X.; Liang, J.; Zhang, K.; Jiang, B.; Luo, H.; Li, L.; An, X.; et al. Visible light-driven Cl-g-C_3N_4 activated peroxydisulfate process for TMP efficient degradation in a wide pH range. *Journal of Water Process Engineering* **2024**, *59*, 105056. DOI: 10.1016/j.jwpe.2024.105056.
